# Chrysanthemum Stunt Viroid Resistance in *Chrysanthemum*

**DOI:** 10.3390/v10120719

**Published:** 2018-12-17

**Authors:** Tomoyuki Nabeshima, Yosuke Matsushita, Munetaka Hosokawa

**Affiliations:** 1 Experimental Farm of Agriculture, Department of Agriculture, Kyoto University, Kizugawa, Kyoto 619-0218, Japan; 2 Institute of Vegetable and Floriculture Science, National Agriculture and Food Research Organization, Tsukuba, Ibaraki 305-0258, Japan; yousuken@affrc.go.jp; 3 Laboratory of Floriculture, Department of Agriculture, Kindai University, Nara, Nara 631-0052, Japan; mune@nara.kindai.ac.jp

**Keywords:** ornamentals, CSVd, chrysanthemum chlorotic mottle viroid, pospiviroidae, crop protection, viroid diagnosis, viroid inoculation, screening

## Abstract

Chrysanthemum stunt viroid (CSVd) is one of the most severe threats in *Chrysanthemum morifolium* production. Over the last decade, several studies have reported the natural occurrence of CSVd resistance in chrysanthemum germplasms. Such CSVd-resistant germplasms are desirable for the stable production of chrysanthemum plants. Current surveys include finding new resistant chrysanthemum cultivars, breeding, and revealing resistant mechanisms. We review the progress, from discovery to current status, of CSVd-resistance studies, while introducing information on the improvement of associated inoculation and diagnostic techniques.

## 1. Introduction

Chrysanthemum (*Chrysanthemum morifolium*) is an economically important ornamental crop and is cultivated throughout the world. Since commercial chrysanthemums are usually propagated vegetatively, virus and viroid diseases are major challenges. One such disease is chrysanthemum stunt, incited by chrysanthemum stunt viroid (CSVd) [1,2,3]. Symptoms of a CSVd infection include stunting, spotted leaves, poor rooting, flower color change, and disturbance of photoperiodic responses related to flowering initiation [4,5,6,7,8,9]. The CSVd is a member of the genus *Pospiviroid,* within the family Pospiviroidae, which has a central conserved region and replicates and accumulates in the nuclei [10]. The CSVd exhibits a 69% sequence identity to the potato spindle tuber viroid (PSTVd), a type species of the family Pospiviroidae, and forms a secondary structure similar to the PSTVd [11]. CSVd can easily be transmitted from plant to plant by sap. The latent infected plants multiply during propagation, and some of them exhibit symptoms during cultivation. CSVd is widely distributed globally, and chrysanthemum stunt disease in chrysanthemum fields has been reported in 26 countries, including 15 in Europe, four in Asia, two in Africa, two in North America, two in Oceania, and one in South America [12].

Attempts for managing chrysanthemum stunt disease have focused on the development of early CSVd detection techniques to eliminate infected plants from the fields, on producing CSVd-free plants [13,14,15,16,17,18,19], and on the development of viroid disinfectants for pruning tools [2,20]. Recently, several studies have demonstrated the existence of resistance in commercial cultivars in Japan [21,22,23,24,25]. Since CSVd and infected plants are widely distributed, and a symptomless alternative host could be the source of the disease [8,26], chrysanthemum production has always been adversely affected by CSVd contamination. Consequently, CSVd resistance, which could withstand unforeseen contamination, would be a desirable trait. Similarly, transgenic approaches for the development of CSVd-resistant plants have been reported [27,28,29]. The transgenic plants is a promising alternative, and the approach is well described in published reviews [30,31,32].

In the present review, we focus on naturally occurring CSVd resistance, its discovery, screening, breeding, and associated inoculation and detection techniques. The use of resistant cultivars and conventional breeding still has key advantages which are associated with their relative ease of application in agricultural production worldwide, due to the restricted use of transgenic crops in Europe. In 1985, two lines of wild potato (*Solanum berthaultii*) were reported to be resistant to PSTVd sap inoculations [33]. PSTVd resistance or tolerance have been reported in other wild potato species [34,35]. However, the resistance traits have not been transferred to cultivated potatoes (*Solanum tuberosum*) [35,36]. In apricot (*Prunus armeniaca*), a screening program with the aim of identifying cultivars resistant to hop stunt viroid was carried out; however, no resistant cultivars were found [37]. Among the various crops suffering from viroid diseases, currently, *C. morifolium* is the only one in which several intentional surveys on viroid resistance have been carried out. Although there are numerous issues that remain unresolved, we believe that our experiences are worth sharing with the research community, since viroid-resistant germplasms are desirable in many crop fields.

## 2. Discovery of CSVd Resistance

Chrysanthemum production has been greatly affected by CSVd and chrysanthemum chlorotic mottle viroid (CChMVd) [38,39,40]. The first step in eradicating this problem is to create viroid-free plants, and several studies have explored this approach [13,14,15,16,17,18,19]. Hosokawa et al. [15,41] established a leaf primordia (LP)-free shoot apical meristem (SAM) culturing method to obtain CSVd- and CChMVd-free plants. They excised LP-free SAMs from infected chrysanthemum plants and put them onto the root tips of cabbages (*Brassica oleracea*), with some of the SAMs growing into viroid-free plants. The next step would be addressing the challenge of re-infection in viroid-free plants, and breeding of resistant cultivars is considered one of the optimal solutions. However, at that time, viroid-resistant chrysanthemum cultivars were not known. Coincidently, the group observed that CSVd was hardly detected in chrysanthemum cultivar samples collected by a local farmer in Fukui prefecture [21]. The cultivar “Utage”, had been cultivated in Fukui for several years, and some of the plants exhibited stunting symptoms. Since “Utage” cultivation had never been subjected to CSVd removal by SAM culturing, the researchers opted to identify the presence of the viroid by RT-PCR and then eliminate CSVd by LP-free SAM culturing. The absence of CSVd in the samples was surprising because, at the time, it was assumed that numerous chrysanthemum cultivars in Japan were already infected by CSVd, and almost all stunting symptoms were attributable to the pathogen. Repeated RT-PCR experiments did not reveal the presence of CSVd, and the results led the researchers to shift from CSVd elimination to CSVd resistance investigation. Subsequently, they inoculated CSVd into five cultivars including “Utage” by grafting and examined the infection status in the scions. They found that the CSVd titer in “Utage” remained low, even 15 weeks after grafting (almost 1/1000 of those in susceptible cultivars). Then, they obtained 67 progenies by self-pollination of “Utage” and conducted grafting experiments again. Among the 67 seedlings, they observed that three plants showed much lower titers of CSVd (1/240, 1/41000, and 1/125000 of that in “Utage”). Although “Utage” and its resistant progenies did not exhibit perfect incompatibilities with CSVd, their resistance was high enough to prevent symptom development. Moreover, CSVd accumulation rates in the plants were quite low, resulting in CSVd titer decreases as the plants grew, suggesting that CSVd contamination would not pose a challenge during their cultivation and propagation. That was the first report of CSVd resistance in chrysanthemums, and it indicated the possibility for CSVd-resistant cultivar breeding.

## 3. Screening of CSVd-Resistant Cultivars

Since the potential of obtaining CSVd resistance from existing cultivars had been suggested, researchers explored more cultivars in the search of CSVd resistance resources. Matsushita et al. [22] grafted 22 lines, including commercial cultivars, wild species, and their hybrids, onto a CSVd-infected susceptible cultivar and found that a cultivar, “Okayama Heiwa”, was resistant to CSVd infection. In “Okayama Heiwa”, CSVd was not detected in the upper leaves 220 days after grafting, while the majority of other cultivars were CSVd-infected 30 days after grafting. Nabeshima et al. [23,24] established a large-scale screening system in which the LP-free SAM were attached to CSVd-infected root tips in the first screening. Subsequently, the plantlets produced stems and leaves, then roots with the specimens (LP-free SAM) genotype. If the cultivar was sensitive or tolerant CSVd it could invade the tips once they fused with the roots and produced embryo-like plantlets. When the SAM has a susceptible genotype, it will be highly contaminated with CSVd and produce CSVd-infected leaves. If the SAM has a resistant genotype, CSVd will not invade it or will disappear after infection, leading to CSVd-free leaves in the plantlets. The small leaves of plantlets were assayed for CSVd by microtissue (MT)-direct RT-PCR [42]. According to the results of the CSVd detection in the upper leaves over a 2 to 4 months of CSVd exposure, the used cultivars were classified into four groups: A. CSVd was detected in all tested leaves with high titers; B. CSVd was detected, but the titers decreased acropetally; C. CSVd was detected, but the titers were low in all the assayed leaves; D. CSVd was not detected during the experimental period [23,24]. CSVd-resistant cultivars could be obtained from groups B, C, and D, although most were from D. This was consistent with long-term cultivation records collected by a chrysanthemum nursery, which listed cultivars that had been the subject of complaints by chrysanthemum growers with regards to the incidence of CSVd disease [24]. By grafting experiments, some CSVd-resistant candidates from groups B and C cultivars were revealed to be CSVd-susceptible, indicating that the screening system using LP-free SAM culturing overestimated the resistance of some cultivars. However, the screening system facilitated the effective screening of large amounts of the assayed cultivars and reduced the number of plants used in the grafting experiment, which was time-consuming and labor-intensive.

In grafting experiments, if a scion remains CSVd-free (or the CSVd titer is below detectable limits) for a long time, it is probable that the scion has some mechanisms that prevent CSVd infection, as reported by Matsushita et al. [22]. However, CSVd presence in the upper leaves of the scion does not always imply susceptibility of the scion, because phloem sap in the upper leaves may contain CSVd-rich sap derived from infected rootstock. Using an in situ hybridization technique, Nabeshima et al. [23] demonstrated that in the shoot tips of “Sei no Issei”, a CSVd-resistant cultivar grafted onto CSVd-infected rootstocks for six months, sieve elements in leaf primordia and unexpanded leaves contained high amounts of CSVd. In such “Sei no Issei” plants, after they were cut from infected rootstocks and allowed to root, the CSVd titers in newly expanded leaves drastically decreased. Similar observations were made in other experiments using “Mari Kazaguruma”, a resistant cultivar [43]. Therefore, in grafting experiments, the decision on whether the specimen is resistant or not has to be made while taking into account the potential of contamination with CSVd molecules directly derived from highly infected CSVd sources. Scions cut from infected rootstocks after adequate exposure (> 2 months) to CSVd are useful for evaluating CSVd resistance characteristics without the effects of fused CSVd-susceptible tissues. This grafting and cutting procedure was adopted in another screening program undertaken by a local public institution in Nara prefecture, and several resistant lines were obtained [25].

By several screening programs, researchers have shown that CSVd resistance exists in commercial chrysanthemum cultivars. Although current screening systems contain labor-intensive processes and some errors, which make the whole screening process much more labor- and time-intensive, this problem will be solved by more efficient inoculation and detection methods, as introduced in the next section.

## 4. Improvements in Inoculation and Detection Techniques

The conventional grafting inoculation method provides stable and high infection ratios. However, it is time-consuming, requires a lot of space, and is labor-intensive. Sap inoculation is easier but is sometimes associated with unstable infection ratios [2]. In recent studies, in vitro transcribed RNA of monomeric sense linear CSVd was shown to be infectious in chrysanthemum plants [44,45]. Furthermore, dimeric sense and antisense head-to-tail linear CSVd were also shown to be infectious in chrysanthemum and provided a rapid test system in combination with agroinfiltration [46]. Such artificial inoculums are attractive alternatives in chrysanthemum CSVd studies due to their sequence uniformity and rapid and high-rates of CSVd infection establishment. For the determination of CSVd infection, molecular detection of CSVd RNA is favorable, because symptom development is sometimes different across cultivars or varied environmental conditions. Here, we introduce a simple detection method, which can skip the RNA extraction step, enabling rapid assays of large numbers of specimens. A reverse transcription loop-mediated isothermal amplification method for CSVd detection has been developed [47,48], which can detect CSVd without thermal cyclers and, therefore, it is more suitable in field studies. A commercial kit (Cycleave ICAN™, Takara, Japan) is also available. For PCR-based methods, MT-direct RT-PCR was developed for a convenient CSVd detection method [42]. The authors used a syringe needle to pierce an infected leaf in order to obtain a minimal amount of crude sap and used it for RT reactions. Therefore, RNA extraction procedures were not required. Recently, MT-direct RT-PCR combined with a one-step SYBR system was adopted for the quantification of CSVd titers [24]. We expect that the combination of the agrobacterium-mediated inoculation system and rapid CSVd detection methods, such as the loop-mediated isothermal amplification method or MT-direct RT-PCR, will enable a more efficient screening of CSVd resistance.

## 5. Possible Mechanisms of CSVd Resistance

The life cycles of pospiviroids include the following steps: entry into the nucleus, nucleoplasm and nucleolus transcribing and processing, exit from the nucleus, cell-to-cell movement, and long-distance movement through the phloem or vascular system [36,49,50,51]. Host factors involved in nuclear entry [52,53], replication, processing [54,55,56,57], and long-distance movement [58,59] have been reported. Viroid resistance could be influenced by factors that negatively affect such viroid–host factor interactions and some mechanisms that massively degrade viroid RNAs. To date, no information on chrysanthemum host factors that influence CSVd is available, and our knowledge on CSVd resistance is limited to inductive reasoning based on observations of CSVd titer changes and localization patterns in inoculated plants.

Nabeshima et al. [43] attempted to outline the factors that are responsible for CSVd resistance in “Mari Kazaguruma”. Through agroinfiltration experiments, they demonstrated that “Mari Kazaguruma”-inoculated leaves accumulated circular CSVd RNA amounts equivalent to those in “Piato”, a susceptible cultivar. However, in the “Mari Kazaguruma”, CSVd systemic infection was not observed even with high amounts of CSVd in the inoculated leaves. They also showed that CSVd distribution, from initial infected cells to neighboring cells, was relatively slow in “Mari Kazaguruma”, suggesting the existence of mechanism(s) that delayed CSVd cell-to-cell movement. Similarly, in other resistant chrysanthemums, unequal distribution of CSVd within a leaf or at the whole-plant level (often observed as acropetal decrease in CSVd titers) was frequently observed, although direct investigations have not been carried out [21,23]. Such phenomena may be explained by low compatibility between host factors and CSVd RNA or the accumulation of chemicals that attenuate viroid distribution, such as callose [60]. RNA silencing that attenuates virus or viroid infection [61,62] is another potential mechanism that could negatively influence CSVd infection. Sano and Matsuura [63] showed that recovery from severe symptoms in PSTVd-infected tomatoes (*Solanum lycopersicum*) occurred alongside sequence-specific RNA degradation. In transgenic studies, RNA silencing mechanisms were applied to confer viroid resistance to tomato [64], *Nicotiana* [65], and chrysanthemum [29]. Di Serio et al. [66] showed that RNA-dependent RNA polymerase 6, which catalyzes the amplification of the double-stranded precursors of secondary small interfering RNAs, has a role in the surveillance mechanism, restraining the entry of PSTVd into the shoot tips of *N. benthamiana*. In susceptible chrysanthemums, CSVd could invade leaf primordia and cells that were very close to the apical dome [21,23] or even the outmost layer of the apical dome [13]. CSVd scarcity in shoot apices, even when CSVd was continuously provided from highly infected rootstocks, was a common characteristic in resistant progenies of “Utage” [21] and resistant cultivars including “Mari Kazaguruma” and “Sei no Issei” [23], which may suggest relatively high CSVd degradation activities in such cultivars. However, the involvement of an RNA-silencing pathway in naturally occurring CSVd resistance has not been examined, and future studies should investigate the phenomenon.

## 6. Breeding

In Japan, some organizations have begun the selection and breeding of resistant commercial chrysanthemums. In a screening program, carried out as part of a research project for establishing CSVd disease management techniques, funded by the Ministry of Agriculture, Forestry, and Fisheries of Japan, from 2010 to 2012, three nurseries participated in the project and subjected their own cultivars to screening. In the program, 420 cultivars were investigated, and 15 resistant cultivars were obtained (partly published in [23,24]). Following the program, local agricultural institutes in Gunma [67], Ibaraki [68], and Miyagi prefecture [69] began searching for resistant sources among their locally cultivated cultivars, although novel CSVd-resistant varieties have not yet been reported. In Nara prefecture, some resistant cultivars have already been identified [25,70], and the resistant cultivars were crossed with each other to obtain new resistant cultivars [71,72].

Matsushita et al. [22] demonstrated the potential of conferring CSVd resistance to various commercial cultivars by crossing resistant and susceptible cultivars. When a resistant cultivar “Okayama Heiwa” was crossed with two susceptible cultivars, “Sei Elza” and “Anri”, which were the pollen parents, 13 of 76 and one of eight plants in F1 progeny were CSVd-resistant, respectively. However, the pattern of inheritance remains unclear. Torata et al. [71] observed that F1 progenies in populations obtained by crossing different resistant cultivars also contained both resistant and susceptible plants, and the ratio of resistant to susceptible F1s was potentially dependent on the resistant cultivars. *C. morifolium* has unstable and variable chromosome numbers that form a hexaploid complex with aneuploidy (2n = 54 ± 7∼10), even within one cultivar, and formation of fragmented chromosomes during mitosis can occur [73]. Furthermore, cultivated chrysanthemums could be classified as segmental allohexaploids [74]. Such high-order polyploid species make genetic analysis challenging. To overcome the challenges associated with complex hybridity and polyploidy, a diploid wild chrysanthemum, *Chrysanthemum seticuspe*, has been used as an alternative model of *C. morifolium*. Some *C. seticuspe* lines have resistant characteristics [75]. Further investigations are required to analyze the hereditary patterns using wild chrysanthemum species.

In summary, in this decade, the number of CSVd-resistant resources is gradually increasing by continuous efforts of some organizations but we are also encountering difficulties in efficient breeding. We will discuss this problem in the last section.

## 7. Future Prospects

As mentioned above, several independent screening and breeding programs have already been initiated in Japan. Researchers can facilitate such efforts by providing improved inoculation, detection systems and developing molecular markers. Moreover, if some genes exhibit different types of resistance, combinations of such genes may confer high resistance against CSVd. Therefore, the continuous search for novel resistant germplasms is critical. Recent advances in sequencing technologies offer great opportunities for searching out target genes in various crops [76,77]. However, mapping of the CSVdresistance gene in *C. morifolium* genome is still a challenging activity because of the plant’s complex genome background and difficulties in crossing material to obtain viable seeds. There are technological improvements that could facilitate selection and breeding programs. In addition, the selection of plant materials that could be manipulated is ongoing, and efforts must be sustained. As described above, utilizing diploid wild species as model plants is an attractive option in the search for CSVd-resistance genes. Recently, the whole genome of a wild species, *C. seticuspe*, has been sequenced [78], and a high-density linkage map for fine-scale quantitative trait locus mapping has been constructed [79]. Future studies will utilize such genomic resources to explore resistance-related genes. Although resistant wild species may not be appropriate breeding materials, information on resistance genes would accelerate the screening of resistant *C. morifolium*. In addition, genome editing techniques could be applied in CSVd-resistance breeding in the future. Recently, clustered regularly interspaced short palindromic repeats (CRISPR)/CRISPR associated proteins-based genome editing was successfully applied to knock out multiple copies of transgenes in *C. morifolium* transgenic lines [80]. Genome editing for the simultaneous modification of homoalleles has been reported in wheat (*Triticum aestivum*) [81], a hexaploid crop. If resistant traits are dependent on cultivar (variety)-specific sequence variants of a few “resistance genes” or “susceptibility genes”, modification of such genes would confer CSVd resistance in existing commercial cultivars.

Notably, previous studies have been carried out under infection with few CSVd strains (or the used strains were not determined). No one can deny the probability that the reported resistance will be overwhelmed by untested CSVd strains, since evidence has shown that even a single nucleotide change may affect replication efficiency [82] and distribution patterns [83,84,85] of viroids in hosts. According to a survey by Yoon et al. [12], at least 117 sequence variants had been deposited in online databases by 2013, but our knowledge on how each variant behaves in *C. morifolium* plants is very limited. To date, several studies have been carried out to reveal viroid population development in field-grown hosts [86,87]. Recent advances in sequencing technologies will provide deeper insights into viroid population dynamics. Which varieties are problematic, how do they occur, and how do they induce symptoms in hosts? Which strains do current resistance sources provide resistance to? Answering such questions would be critical in facilitating the utilization of limited resistant sources, through the combination of multiple resistant genes to produce durable resistance or the establishment of chrysanthemum stunt disease management systems.
